# How to attract young talent to nuclear medicine step 1: a survey conducted by the EANM Oncology and Theranostics Committee to understand the expectations of the next generation

**DOI:** 10.1007/s00259-023-06389-9

**Published:** 2023-09-09

**Authors:** Valentina Ambrosini, Sofia Carrilho Vaz, Niloefar Ahmadi Bidakhvidi, Marion Chanchou, Matthijs C. F. Cysouw, Francesca Serani, Conrad-Amadeus Voltin, Francoise Kraeber-Bodere, Christophe M. Deroose, Lioe-Fee De Geus-Oei, Matthias Eiber, Gopinath Gnanasegaran, Martin Gotthardt, Carsten Kobe, Mark W. Konijnenberg, Cristina Nanni, Daniela E. Oprea Lager, Kambiz Rahbar, David Taieb, Felix M. Mottaghy, Karolien Goffin, Ken Herrmann

**Affiliations:** 1 Nuclear Medicine, IRCCS Azienda Ospedaliero-Universitaria Di Bologna, Via Massarenti 9, 40138 Bologna, Italy; 2 Nuclear Medicine, Alma Mater Studiorum University of Bologna, Bologna, Italy; 3 Nuclear Medicine-Radiopharmacology, Champalimaud Clinical Center, Champalimaud Foundation, Lisbon, Portugal; 4 Nuclear Medicine, University Hospitals Leuven, Leuven, Belgium; 5 Nuclear Medicine and Molecular Imaging, Department of Imaging and Pathology, KU Leuven, Leuven, Belgium; 6 University Hospital Assistant in Biophysics and Nuclear Medicine, Jean Perrin Cancer Center, Clermont Auvergne University, UMR 1240 INSERM/IMoST UCA, Clermont-Ferrand, France; 7 Department of Radiology and Nuclear Medicine, Location VUmc, De Boelelaan 1117 1081 HV, Amsterdam, The Netherlands; 8 Department of Nuclear Medicine, Faculty of Medicine and University Hospital Cologne, University of Cologne, Cologne, Germany; 9 INSERM, CNRS, CRCI2NA, Médecine Nucléaire, Nantes Université, Université Angers, CHU Nantes, F-44000 Nantes, France; 10 Department of Radiology, Leiden University Medical Center (LUMC), Leiden, The Netherlands; 11 Biomedical Photonic Imaging Group, University of Twente, Enschede, The Netherlands; 12 Department of Radiation Science & Technology, Delft University of Technology, Delft, The Netherlands; 13 Department of Nuclear Medicine, Technical University Munich, Klinikum Rechts Der Isar, Munich, Germany; 14 Department of Nuclear Medicine, Royal Free London NHS Foundation Trust, London, UK; 15 Department of Medical Imaging, Radboudumc, P.O. Box 9101, 6500 HB Nijmegen, The Netherlands; 16 Radiology & Nuclear Medicine Department, Erasmus MC, Rotterdam, The Netherlands; 17 Department of Radiology and Nuclear Medicine, Amsterdam University Medical Centers, Vrije Universiteit Amsterdam, Amsterdam, The Netherlands; 18 Department of Nuclear Medicine, University Hospital Muenster, Muenster, Germany; 19 Nuclear Medicine Diagnostic Imaging and Endoradiotherapy Center Aix-Marseille University CHU de La Timone, Marseille Cedex 5, Marseille, France; 20 Department of Nuclear Medicine, University Hospital RWTH Aachen University, Aachen, Germany; 21 Department of Radiology and Nuclear Medicine, Maastricht University Medical Center (MUMC+), Maastricht, The Netherlands; 22 Department of Nuclear Medicine, University of Duisburg-Essen and German Cancer Consortium (DKTK)-University Hospital Essen, Hufelandstr. 55, 45147 Essen, Germany

The professional non-profit medical European Association of Nuclear Medicine (EANM) was founded in 1985. Nuclear medicine (NM) was recognized by the “Union Européenne des Médecins Spécialistes” (European Union of Medical Specialists–UEMS) as an independent medical specialty in 1989. Since then, there has been an increasing number of nuclear medicine physicians all over Europe.

Last year, during a meeting of the EANM Oncology and Theranostics Committee (OTC), an active discussion took place on strategies to attract and promote the involvement and participation of young generations in NM. When compared to the previous generations, in some countries, it seemed that residents are attracted to clinical work rather than research/academia or abandoning NM training for another specialty.

Moreover, work force shortages in Europe (https://www.ela.europa.eu/en/news/labour-shortages-europe-labour-market-tightening) also affect the medical sector with the World Health Organization projecting a shortfall of 10 million health workers by 2030 (https://www.who.int/health-topics/health-workforce#tab=tab_1). NM and even larger imaging sub-specialties such as radiology are affected (https://www.acr.org/Practice-Management-Quality-Informatics/ACR-Bulletin/Articles/March-2022/The-Radiology-Labor-Shortage), triggered by both an increased demand and an ageing work force. In Germany, the percentage of board-certified NM physicians aged over 60 and 65 years increased tremendously from 2003 to 2021 (from 16.3 and 1.5 to 27.0% and 9.5%, respectively). Firstly, this shortage of young talents affects the delivery of clinical service. Secondly, the younger generations’ participation in research is also a fundamental pre-requisite to promote and support the rapidly evolving field of NM. Notwithstanding different training programs across countries (e.g., NM is integrated with radiology residency in some countries), the number of residents who choose NM as a specialty has been declining in many countries in the past few years [1]. Analysis of the 2017 American College of Radiology Commission on Human Resources Workforce Survey shows on one hand an increase in the NM/nuclear-radiology (NR) physicians hire over the 2014–2017 period, but on the other hand reports a decrease in the number of traditional NM residencies and NR fellowships (79 to 58 programs) and in the combined number of NM and NR trainees (173 to 82 trainees) [1]. Furthermore, there was a decrease of approximately 7% in the number of first-year ACGME (Accreditation Council for Graduate Medical Education) Residents and Fellows in Radiology and Diagnostic Radiology between 2016 and 2021 (https://www.aamc.org/data-reports/workforce/data/percentage-change-first-year-acgme-residents-fellows-specialty-2016-2021).

The potential reasons for the increasing difficulty in attracting medical students to the field of diagnostic imaging are multi-factorial: different expectations from younger generations (e.g., more attentive to pursuing a good quality of life as compared to their predecessors; a possible perceived decline of professional recognition), cultural/society changes (e.g., more challenging family structures and involvement in non-medical activities), a different relationship with patients that challenges the doctor/patient mutual trust (e.g., need of malpractice insurance, pressure, and criticism among medical professionals and patients), a mutated relationship between doctors and institutions (e.g., increase of the request to solve/manage bureaucratic issues, debatable remuneration in view of the amount of working hours, often insufficient workforce capacity), competition/stress to achieve higher scientific impact factor, poorly equipped departments (e.g., complex liaison with basic research, insufficiently equipped radiopharmacy with only few accessible radiopharmaceuticals, lack of medical physicist for radiation protection and dosimetry); deficient working conditions (e.g., absence of digital clinical process where data are easily collected, lack of data manager/statistician for big data analysis and/or medical librarian for accurate literature search), and lack of a clear career path (e.g., lack/uncertainty of professional progression, lack of new challenges to keep motivation).

The first step to potentially solve such a problem is a better understanding of the challenges, needs, and shortfalls of the current set up. To find ways to objectify young colleagues’ participation in research and active involvement in activities of the European Association of Nuclear Medicine (EANM), the EANM Oncology and Theranostics Committee (OTC) with the support of the EANM Board promoted a survey addressing current NM residents from countries belonging to the EANM National Member Societies to explore their points of view on the perceived needs and expectations of younger generations.

A set of 18 questions were formulated within the EANM OTC. After approval by the EANM committee coordinator, the survey (Table 1) was considered suitable for an online assessment of NM residents’ expectations and perceived needs for their peers to enhance their participation in research and activities of EANM.

**Table 1:** Survey among current European residents in nuclear medicine

Q1	Age (years old)
Q2	Year of residency
Q3	Country
Q4	Have you ever wished to be more involved in the association activities? (yes/no)
Q5	If you answered yes to question 4, you wish to be involved
	In EANM activities
	In ESMIT courses (preparation of material and/or presentations during in presence events)
Q6	If you answered yes to question 5, please indicate in what activities (free text)
Q7	Are you an author of at least one published paper in a peer-reviewed journal? (yes/no)
Q8	If you answered yes to question 7, are you first author on at least one of these papers? (yes/no)
Q9	Are you currently involved in at least one research project/area at your center? (yes/no)
Q10	Would you be willing to dedicate more of your free time to EANM activities? (yes/no)
Q11	When you picture yourself in 10 years time, you wish your work-day would include:
	*A mix of clinical + research (even if research will likely be performed at least in part in your free time)*
	100% patients' care
	100% research activity
Q12	You are interested in
	Diagnostic nuclear medicine
	Therapeutic nuclear medicine
	Both
Q13	How would you suggest to promote young peoples involvement in the association activities?
	I would like to be involved in planning EANM annual congress activities dedicated to residents
	I would like to be part of projects but I would need someone to mentor me
	Increase number of residents-oriented events (how to: do statistics; design a study; write an effective paper)
	Other
Q14	If you answered "other" to question 13, please insert your suggestions below (free text)
Q15	What do you expect from the EANM as a young professional? (free text)
Q16	Would you like to participate to a recognized academic exchange or in an internship outside your country? (yes/no)
Q17	If you answered yes to question 16, you would like to participate to
	A recognized academic exchange in Europe
	A recognized academic exchange outside Europe
	An internship in Europe
	An internship outside Europe
Q18	If you answered yes to question 16, would you tell us why? (free text)

The prospective anonymous survey was created in a Google module, and the corresponding link was shared through the OTC EANM social media channels (LinkedIn and Facebook), by personal contact of the EANM National Member Societies (who distributed the survey within the national NM societies), by direct involvement of the representatives of residents’ national associations (when available) and by direct involvement of the co-authors of this manuscript. The link was accessible from March 28^th^ 2023 until May 18^th^ 2023. During this time, reminders were published on the OTC webpage and other social media platforms. Both quantitative and qualitative data were collected and analyzed using descriptive statistical methods and thematic analysis, respectively.

Overall, 190 respondents completed the online anonymous survey. Slightly more than half of respondents (52.1%) were ≥ 31 years old (with 28% of the entire cohort ≥ 33 years old), while 47.4% were ≤ 30 years old (Fig. 1A; Q1). Respondents were evenly distributed among the different years of residency (approximately 20% in each of the 1^st^ to 4^th^ year; 15% in the 5^th^ year, not present in every country; Fig. 1B; Q2). Respondents were coming from all over Europe, representing more than 20 different countries, with Italy (14.2%), Germany (9.5%), Sweden (7.9%), Belgium (7.4%), and Romania (7.4%) being the most frequent (Fig. 1C; Q3). Notably, these percentages are obtained for the entire group of respondents and are not corrected for the total number of NM residents per country.

**Fig. 1:**
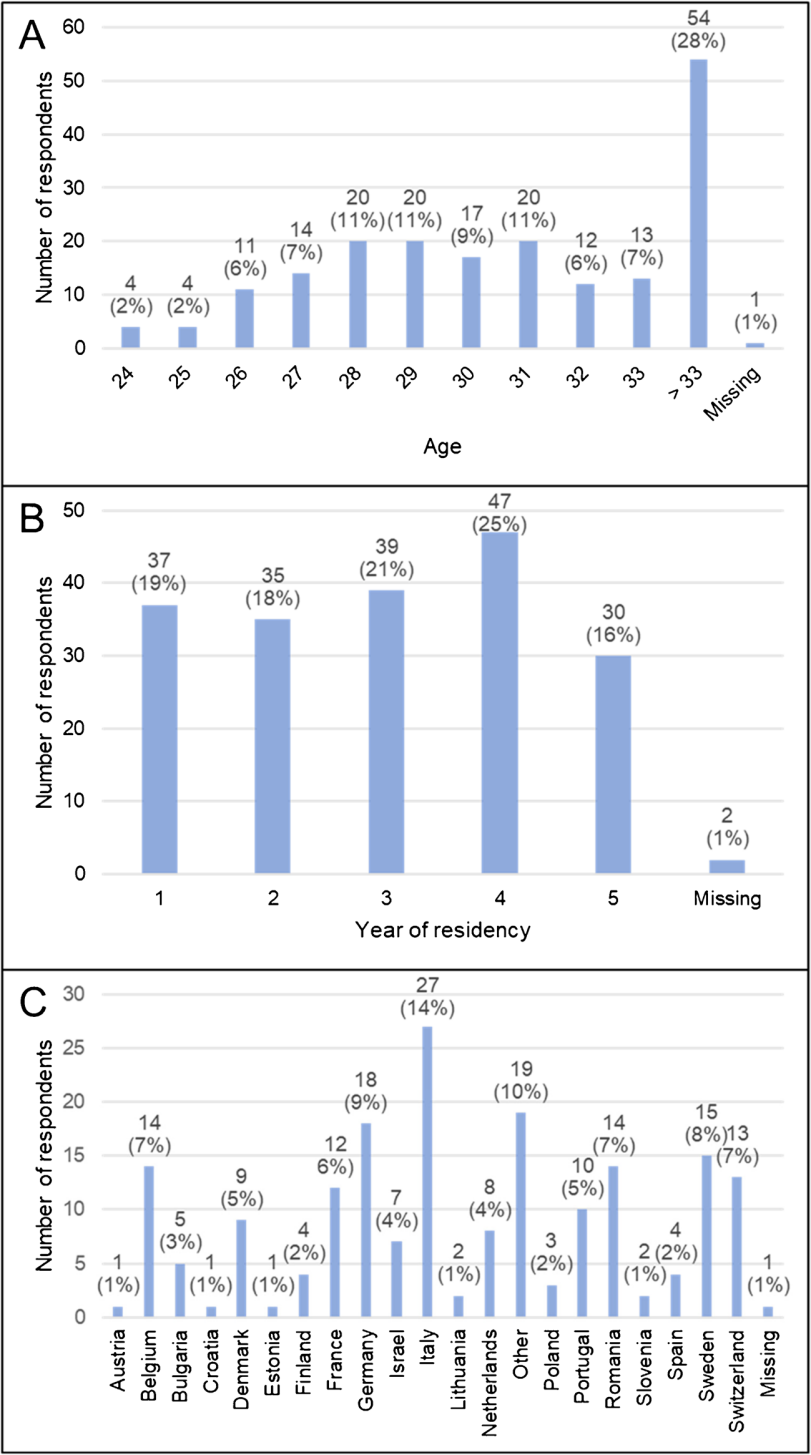
Respondents background (Q1–Q2–Q3). The number of respondents according to their age (**A**), year of residency (**B**), and country (**C**) are presented (percentages are expressed without decimals)

The majority of respondents expressed interest to be more involved in activities of the association (111/190, 58.4%), especially in EANM (69.4%,) followed by the European School of Multimodality Imaging & Therapy (ESMIT) (29.7%) (Fig. 2; Q4, Q5). More than 60% of respondents want to dedicate more of their free time to EANM activities (Q10; 118/190, 62%). Their interest (Fig. 2; Q6; results categorized from free text) was mostly oriented towards participation in educational activities (workshops, mentoring, events that promote the interaction with other residents, the EANM annual conference), followed by participation into research projects. A minority was interested in being involved in EANM committees. The proportion of respondents interested in being more involved in the association activity varied widely among countries (only reporting countries with ≥ 5 respondents: 86% for Israel (6/7) and Romania (12/14), 63% for the Netherlands (5/8), 62% for Switzerland (8/13), 61% for Germany (11/18), 52% for Italy (14/27), and 50% for Belgium (7/14).

**Fig. 2:**
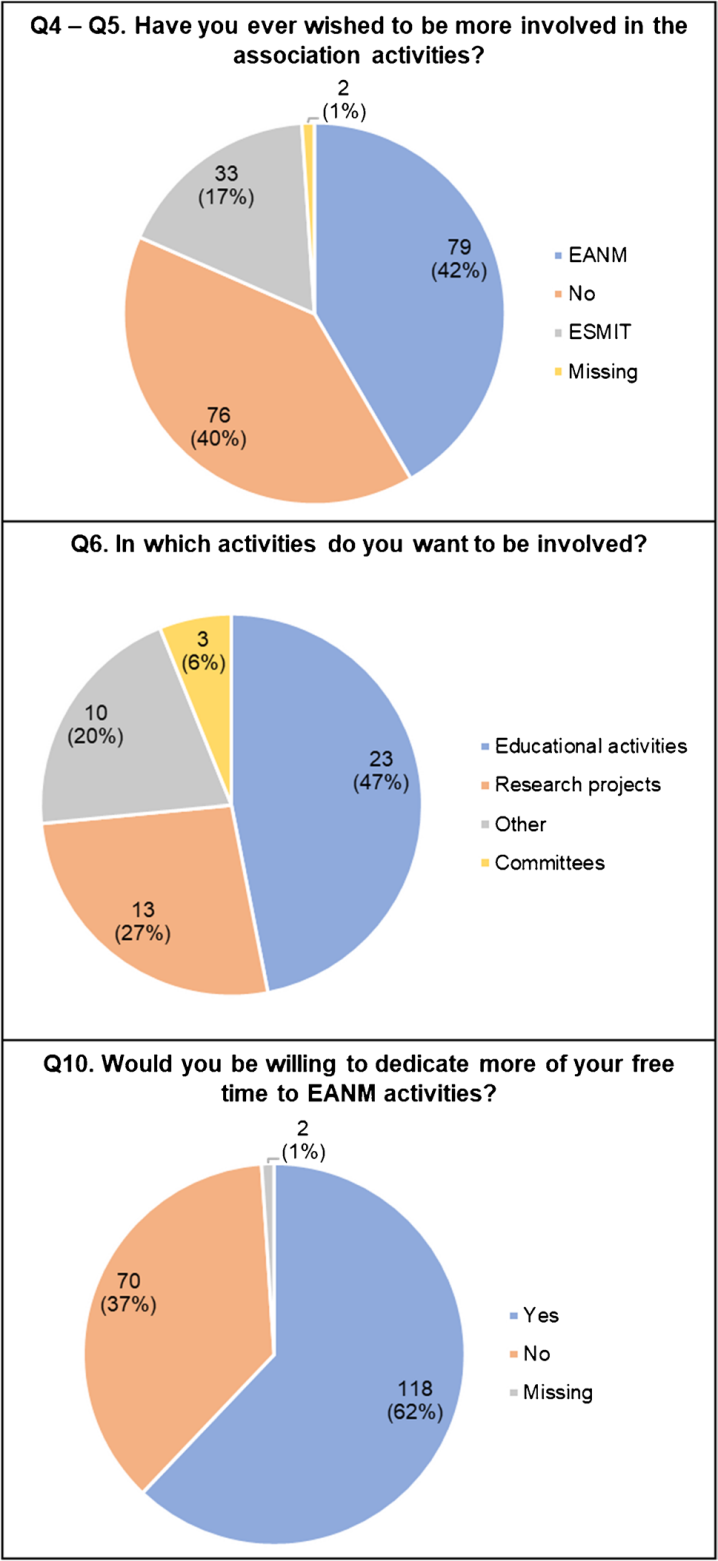
Involvement in the association (Q4–Q5–Q6–Q10)

Slightly more than half of the respondents (52.6%) are authors of at least one published paper in a peer reviewed journal (Fig. 3; Q7), in most cases as first author (Q8; 67/100, 67%). Sixty percent of respondents were also involved in at least one research project/area at their center (Q9).

**Fig. 3:**
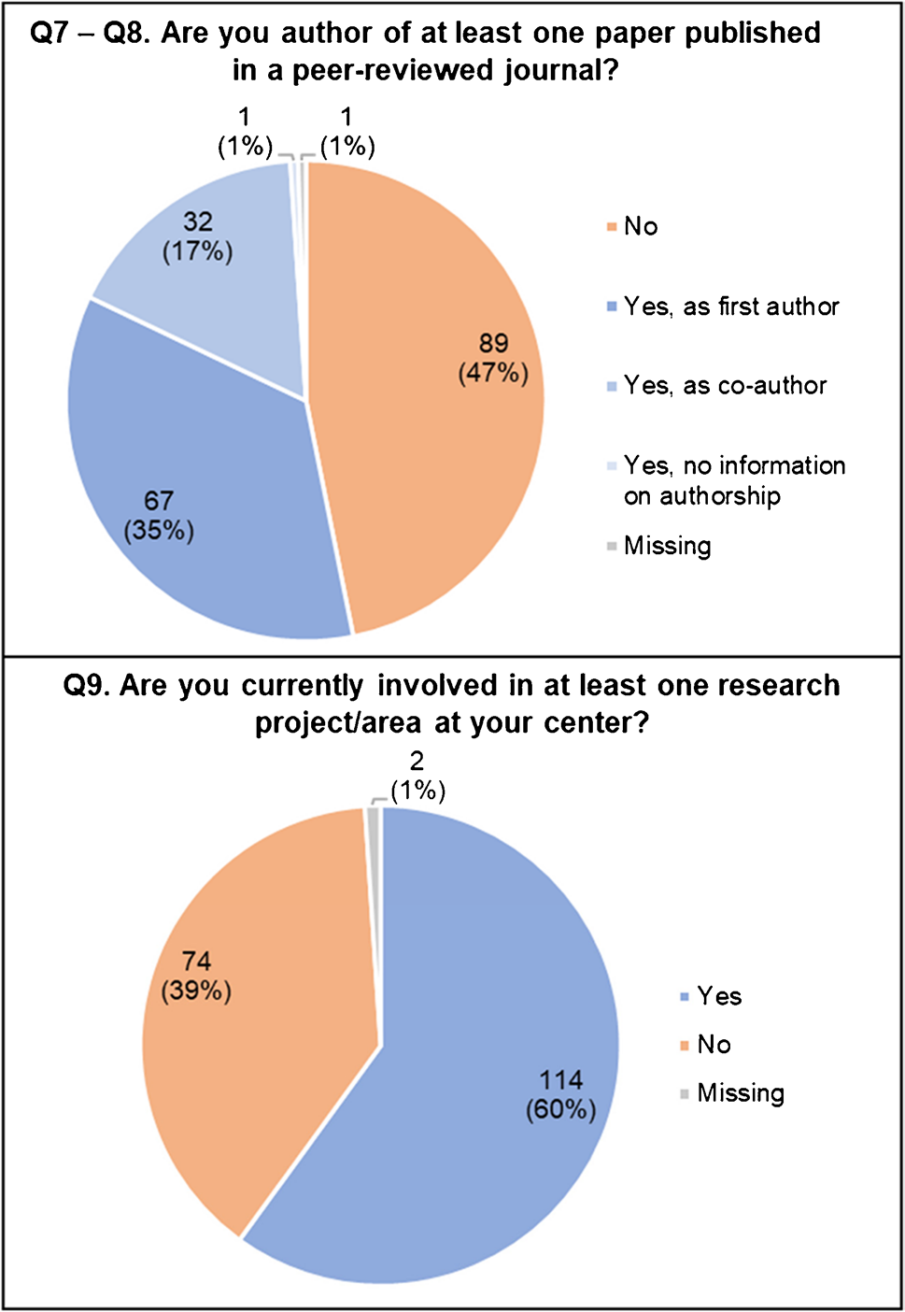
Respondents’ scientific background (Q7–Q8–Q9)

When asked in which professional environment they would picture themselves in 10-years’ time (Q11, Fig. 4), most residents (143/190, 75.3%) answered they wished their future work would include a mix of clinical and research activities (even if research would likely need to be performed, at least in part, in their free time), followed by 40/190 (21.1%) who wished to be dedicated only to clinical work and a minority (3/190, 1.6%) to research only. The respondents’ interest (Q12, Fig. 4) was mainly focused on theranostic NM (diagnostic plus therapeutic, 152/190, 80%) followed by diagnostic NM only (32/190, 16.8%) and therapeutic NM only.

**Fig. 4:**
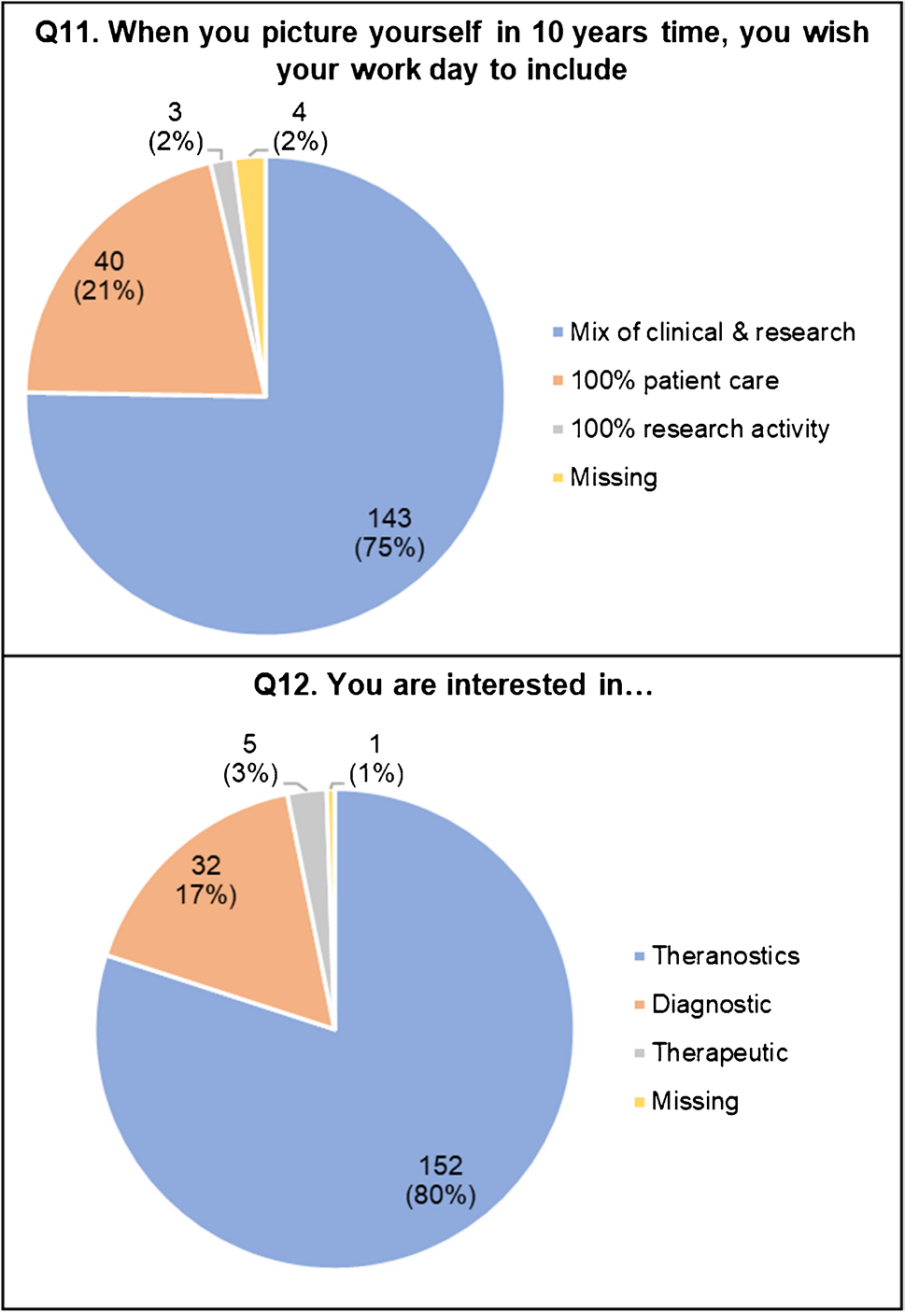
Respondents’ future expectations (Q11–Q12)

When asked to provide suggestions on how to promote young people participation to the association activities (Q13, Table 2), the two most frequent indications were to increase the number of resident-oriented events (77/190, 40.5%; e.g., how to do statistics, how to design a study, how to write an effective paper, etc.) and to have mentoring programs (68/190, 35.8%), while 25/190 (13.2%) wished to be directly involved in the planning of the annual conference events dedicated to residents. Among the 13/190 (6.8%) of respondents who answered “other” to Q13, 7/13 provided a full text description (Q14) of the actions needed in their perspective: suggestions included to favor mentoring, interaction among residents, lower conference fees; one respondent suggested that the involvement in research should be monetized.

**Table 2:** Residents’ expectations and needs

Q13. How would you suggest to promote young people involvement in the association activities?		
	*n*	%
Increase number of residents-oriented events (how to: do statistics; design a study; write an effective paper)	77	40.5
I would like to be part of projects but I would need someone to mentor me	68	35.8
I would like to be involved in planning EANM annual congress activities dedicated to residents	25	13.2
Other	13	6.8
Missing	7	3.7
Q14: if answering “Other” to Q13		
No suggestion	6	46.2
Free text	7	53.8

Young professionals’ expectations from EANM were provided as free text (Q15, Table 3) by 78/190 (41%) respondents. Answers were categorized in Table 3 into a few macro areas with educational programs (49%, including more practical courses), high-quality publications/guidelines and research (14%), guidance for studying and networking (10%), and resident-oriented events (10%) being the most frequent. Some respondents asked for more accessible (lower) rates dedicated to residents.

**Table 3:** Residents’ expectations from EANM

Q15. What do you expect from the EANM as a young professional? (categorized from free text answers)		
	*n*	%
Education	38	48.7
High quality publication and research	11	14.1
Guidance on study/networking opportunities	8	10.3
Residents event	8	10.3
Education and interaction	5	6.4
Mentoring	2	2.6
No expectation	2	2.6
Enhance participation in research	1	1.3
Increase the number of residents oral ppt	1	1.3
Interaction	1	1.3
Coordination of the big changes in NM	1	1.3
Total	78	100.0

Most respondents were willing to take part to an exchange program or an internship abroad (Q16; Table 4; 143/90, 75.3%), mostly as part of a recognized academic exchange in Europe (Q17; 57/143, 30%), an internship in Europe (42/143, 22.1%), or outside Europe (equally distributed between academic recognized 23/143 and internship 23/143, 21.1%). On the contrary, 45/190 (23.7%) respondents were not interested in going abroad. Reasons (Q18, Table 4) in favor of taking part to a working period abroad were provided as free text by 83/190 respondents (43.7%): the most frequent reason was to increase their professional experience (on procedures, working standards, different protocols, broaden practical skills, be exposed to a wider variety of clinical cases; 86%).

**Table 4:** Respondents’ opinion regarding studying abroad

Q16. Would you like to participate to a recognized academic exchange or in an internship outside your country?		
	*n*	%
No	45	23.7
Yes	143	75.3
Missing	2	1.1
Q17: if answered “yes” to Q16	*n*	%
A recognized academic exchange in Europe	57	30.0
A recognized academic exchange outside Europe	23	12.1
Internship in Europe	42	22.1
Internship outside Europe	23	12.1
Missing	45	23.7
Q18. If you answered yes to question 16, would you tell us why? (free text)		
	*n*	%
Free text suggestions	83	43.7
Missing	107	56.3
Q18: if answered “yes” to Q16*		
	*n*	%
Different procedures	2	2.4
Experience	71	85.5
Experience and interaction	2	2.4
Experience/education	1	1.2
Education	1	1.2
Culture	2	2.4
Other	4	4.8

*Categorized from free text answers

There is a tremendous need to attract young talents to NM since we are facing an increased demand for specialists needed to both replace the aging work force and to cover the increasing demands for NM diagnostic procedures (PET/CT is included in the routine work-up of most tumours) and theranostic applications. Younger generations represent the future of research, in medicine in general and in NM in particular. NM can be considered a rather small and fairly “newborn” specialty (as compared to internal medicine, surgery or cardiology). However, the advances in the past two decades have completely changed perception of NM is by physicians, the public/society, medical students, and residents. The introduction of hybrid imaging, with computed tomography (CT) and magnetic resonance imaging (MRI) led to the overall improvement of diagnostic sensitivity and specificity. The advent of [^18^F]FDG ([^18^F]fluoro-2-deoxy-D-glucose) PET/CT (positron emission tomography) followed by the many different positron-emitting radiopharmaceuticals led to the incorporation of PET/CT in the routine diagnostic flow-charts of most tumours. More recently, the introduction of theranostics into clinical practice (e.g., neuroendocrine tumors and prostate cancer) may have the potential to attract the interest of the younger students who wish to keep a close contact with patients’ and their management, as compared to the diagnostic setting alone.

Since NM is a rapidly evolving field, strictly linked to the development and clinical use of new radiopharmaceuticals or radioactive devices for both diagnosis and therapy, all efforts should be made to increase the participation of the younger generation in research. Many academic programs, conferences, and web resources are available, probably even more today than at the time most of the authors of this paper were young students themselves; however, younger generations may find it difficult to approach and find their “place” in research.

This online anonymous survey included a set of 18 questions answered by 190 current NM residents, with five countries having the higher response rates (Italy, Germany, Belgium, Sweden, and Romania). The survey generated 5 key findings: (1) overall, 60% of respondents want to increase their involvement in EANM (EANM > ESMIT), however with high variability between countries; (2) 60% of respondents are currently involved in research, which has often resulted in participation to a paper published in a peer-review journal; (3) for their future work situation, respondents have a strong preference to combine clinical work with research activities and be active in the broad field of theranostics (imaging and therapy); (4) in order to enhance their careers, respondents mainly ask for more resident-oriented events, mentoring programs, guidance for studying and networking, and educational programs/guidelines; and (5) 75% of respondents want to participate in an exchange program to enhance their professional experience.

Interestingly, theranostics was indicated as the most appealing aspect of NM. On one hand, this result is expected in view of the rapidly evolving research in this field and of the exponential increase in the number of centres activating theranostic facilities, mostly following the initial approval of [177Lu]Lu-DOTATATE in 2019 and the recent advances in prostate cancer theranostics that are expected to further dramatically increase the demand and need of theranostic specialists. On the other hand, it shows that younger generations are willing to be part of the new era of patients care in which the NM physician is an active member of the multidisciplinary team of treating physicians.

When asked how they would suggest to promote young people involvement in the association activities, about 40% were willing to attend residents-oriented events. Respondents were interested in attending events to promote and enhance their knowledge on how to do research (e.g., how to do statistics, design a study, and write an effective paper), which probably underlines the suboptimal representation of specific research-oriented courses within the different academic programs. Since teaching of statistics and methodology are in fact present in most specialties and medical training programs, it may be speculated that there is a need for more practically-oriented courses that can teach how to deal with the different steps and difficulties of real-life research (e.g., study design, ethical committee approval, data collection/analysis, and paper writing). Another interesting point from the survey is that approximately 36% of respondents expressed willingness of being mentored. This may indicate the need of having someone/supervisor outside/inside the department to support and challenge the resident, maybe enabling to facilitate some institutional/organizational difficulties and providing an independent way of exchanges of ideas and a possibility for brainstorming. Curiously, a minority of respondents was interested in being involved into committees’ activity. This may reflect a lack of awareness of the committees’ role and duties within EANM. It can also indicate the need of seniors mentoring the younger colleagues that will be involved in the committees in the future.

In the present cohort, respondents expect from EANM primarily to promote educational activities, to provide guidance on study/networking and to be a reference for guidelines and research. This result was unexpected since a lot of EANM efforts (including ESMIT, Continuing Medical Education (CME) courses, and Learn & Improve Professional Skills (LIPS) Track courses during the annual conference) are already dedicated to promote educational activities, including free weekly webinars. The percentage of residents being members of the EANM remains to be clarified. Usually, this would give them full access to several educational formats available, both on-line and on-site.

In the future, it may be considered to enhance the social media promotion of such already existing education activities organised by EANM and ESMIT in order to reach a wider potential audience. Moreover, a number of events dedicated to train residents for a future career in research could also be thought of to integrate with more practical workshops the more formal academic programs.

Since two-thirds of respondents were willing to take part to an academic exchange/internship abroad, mainly as a recognized academic exchange within Europe to broaden their medical experience, every effort should be made in the future to enhance and facilitate residents’ experiences abroad to increase their knowledge on different procedures, working standards, different protocols, to broaden practical skills and the exposure to a wider variety of clinical cases.

The results of the survey however need to be interpreted with caution, since they are potentially biased by different factors. The promotion of the survey might have been higher in certain countries, even thanks to the help of national residents’ associations (where available). Additionally, the respondents may be more motivated compared to those who are not actively involved yet, as indicated by the fact that most respondents were authors of at least one paper in a peer-reviewed journal and were involved at least one research activity/project at their center. This could introduce a selection bias in the respondents’ pool.

It is also relevant to notice that the proportion of residents interested in being more involved in the association activities is variable among countries (only reporting countries with ≥ 5 respondents: 86% for Israel (6/7) and Romania (12/14), 63% for the Netherlands (5/8), 62% for Switzerland (8/13), 61% for Germany (11/18), 52% for Italy (14/27), and 50% for Belgium (7/14). A rationale for this variability could not be extracted from the current survey. Respondents from all years of residency were represented relatively evenly (with expectedly fewer respondents from the 5^th^ year of residency, not present in all countries), indicating that they are all concerned about their training and future opportunities. Quite unexpectedly given the duration of academic degree courses in medicine, most respondents were older than 31 years of age, with 28% of the entire cohort being older than 33 years. This may be due to different reasons including the possibility that some students graduated later, entered nuclear medicine after another course/specialty, extended the specialty training period (e.g., pregnancy leave during specialty) or performed a research track during residency.

To face the increasing demand of NM specialists and to support the scientific development of NM, it is mandatory to both enhance younger generations involvement in research and to attract young medical students to choose NM as a specialty. It is thrilling to see that younger generations are interested in a career in NM, mainly as a combination of patients’ care and research. Given the many existing events (even free and online), it is surprising that the most substantial request by current residents is to enhance education activities. However, at a more attentive look, this likely reflects the demand of more practically oriented courses, including events to build and provide the background knowledge needed to support and develop a research career.

## Data Availability

The datasets generated during and/or analyzed during the current study are available from the corresponding author on reasonable request.
